# Gene Therapy Targeting *PCSK9*

**DOI:** 10.3390/metabo12010070

**Published:** 2022-01-12

**Authors:** Julius L. Katzmann, Arjen J. Cupido, Ulrich Laufs

**Affiliations:** 1 Department of Cardiology, University Hospital Leipzig, 04103 Leipzig, Germany; Ulrich.Laufs@medizin.uni-leipzig.de; 2 Department of Vascular Medicine, Amsterdam University Medical Centers, location AMC, 1105 AZ Amsterdam, The Netherlands; a.j.cupido@amsterdamumc.nl; 3 Department of Cardiology, Division Heart & Lungs, University Medical Center Utrecht, Utrecht University, 3584 CX Utrecht, The Netherlands; 4 Division of Cardiology, Department of Medicine, University of California, Los Angeles, Los Angeles, CA 90095, USA

**Keywords:** PCSK9, hypercholesterolemia, cardiovascular disease, LDL cholesterol, gene editing, base editing, CRISPR/Cas, in vivo

## Abstract

The last decades of research in cardiovascular prevention have been characterized by successful bench-to-bedside developments for the treatment of low-density lipoprotein (LDL) hypercholesterolemia. Recent examples include the inhibition of proprotein convertase subtilisin/kexin type 9 (PCSK9) with monoclonal antibodies, small interfering RNA and antisense RNA drugs. The cumulative effects of LDL cholesterol on atherosclerosis make early, potent, and long-term reductions in LDL cholesterol desirable—ideally without the need of regular intake or application of medication and importantly, without side effects. Current reports show durable LDL cholesterol reductions in primates following one single treatment with *PCSK9* gene or base editors. Use of the CRISPR/Cas system enables precise genome editing down to single-nucleotide changes. Provided safety and documentation of a reduction in cardiovascular events, this novel technique has the potential to fundamentally change our current concepts of cardiovascular prevention. In this review, the application of the CRISPR/Cas system is explained and the current state of in vivo approaches of *PCSK9* editing is presented.

## 1. Introduction

Atherosclerotic cardiovascular disease remains the leading cause of death worldwide. A major contributor to the development and progression of atherosclerosis are elevated low-density lipoprotein cholesterol (LDL-C) concentrations. Patients with familial hypercholesterolemia (FH) are characterized by lifelong exposure to high LDL-C that causes premature atherosclerotic cardiovascular disease [1]. FH is a highly prevalent genetic disorder with a prevalence of 1:300 [2]. Mutations in two genes—*LDLR* and *APOB*—were known to cause FH, until in 2003, mutations of a third gene were identified to cause FH, i.e., *PCSK9*, encoding proprotein convertase subtilisin/kexin type 9 (PCSK9) [3]. This clinical observation was the starting point of a rapid translation from “bench-to-bedside” of different approaches to therapeutically inhibit PCSK9 function which led to the development and clinical use of monoclonal antibodies against PCSK9 [4]. Two fully human antibodies, evolocumab and alirocumab, were shown to lower LDL-C and cardiovascular risk in large cardiovascular outcome trials [5]. The antibodies are administered every two or four weeks. A novel approach using the small interfering RNA (siRNA) inclisiran shows similar efficacy on LDL-C lowering, and a trial testing the effect on cardiovascular outcomes (ORION-4) is currently ongoing. The RNA drugs target the PCSK9 pathway intracellularly at an earlier step compared to the extracellular inhibition of the antibodies. After a loading dose, the siRNA inclisiran needs to be administered twice a year.

Current research has gone one step further: only recently, successful gene editing of *PCSK9* has been shown to durably lower LDL-C in primates. This approach of addressing PCSK9 at the gene level has the potential of a sustained effect after one single application.

It is premature to draw conclusions on the use of this approach in humans. However, the genetic and clinical evidence that naturally occurring variants in *PCSK9* are associated with markedly reduced cardiovascular risk and not with adverse health effects provides a rationale to target PCSK9 at the gene level. It furthermore deserves mention that—provided long-term efficacy and safety—this “once-and-done” [6] approach would overcome one of the most important obstacles of prevention, i.e., the long-term adherence to lifestyle changes and medications.

In this review, we provide an overview on therapeutic targeting of PCSK9, an introduction to the mechanisms and potential applications of gene and base editing especially by exploiting the clustered regularly interspaced short palindromic repeat (CRISPR)/CRISPR-associated (Cas) system, and discuss the current state of the literature on editing *PCSK9* in vivo.

## 2. PCSK9, LDL-C, and Atherosclerotic Cardiovascular Disease

The involvement of PCSK9 in lipoprotein metabolism was discovered in 2003 [3]. The mechanism how PCSK9 is involved in the regulation of LDL-C concentration is mediated by the binding of extracellular-circulating PCSK9 with the LDL receptor on the hepatocyte surface. If the LDL receptor is internalized after removing an LDL particle from circulation, and PCSK9 is bound to this LDL receptor-LDL particle complex, the LDL receptor is directed to lysosomal degradation. As a consequence, LDL uptake is diminished because of a lower abundance of LDL receptors on the hepatocyte surface, and LDL-C concentration increases [7,8]. Internalized LDL receptors without PCSK9 bound to it are recycled back to the hepatocyte surface, resulting in a higher abundance of LDL receptors and thus an increased capacity for removing LDL particles from circulation. Individuals with loss-of-function mutation in *PCSK9* have lower LDL-C and up to 88% lower risk of coronary artery disease [9]. Homozygous inheritance of *PCSK9* loss-of-function mutations does not appear to be associated with adverse health issues, despite very low LDL-C serum concentrations [10,11]. These observations provided a strong rationale to develop therapeutic approaches to inhibit PCSK9 function. Apart from lowering LDL-C and by this, preventing the progression of atherosclerosis, possible direct effects of PCSK9 on atherosclerosis and its involvement in inflammation, triglyceride-rich lipoprotein metabolism, platelet activation, cancer, and viral infections have been described [12,13].

The use of fully human monoclonal antibodies soon emerged as a promising strategy to inhibit PCSK9 function [14]. After initial trials showed lowering of LDL-C by about 60% with good tolerability, two large cardiovascular outcome trials with the antibodies evolocumab and alirocumab were conducted. Both the Further Cardiovascular Outcomes Research with PCSK9 Inhibition in Subjects with Elevated Risk (FOURIER) [15] and the ODYSSEY Outcomes study [16] demonstrated a 15% relative reduction in cardiovascular risk on top of statin therapy during 2.2 and 2.8 years of follow-up, respectively.

Apart from neutralizing the targeted protein by monoclonal antibodies, another option is targeting the messenger RNA (mRNA) encoding the respective protein. The two main classes of RNA-targeting drugs are siRNAs and antisense oligonucleotides (ASOs). siRNAs are double-stranded mRNA molecules. The so-called guide strand contains a complementary sequence to the target mRNA sequence. After binding the complementary mRNA, the RNA-induced silencing complex gets assembled and leads to the cleavage of the target mRNA. Importantly, the guide strand is not cleaved, and one single guide strand can facilitate cleavage of several mRNAs, likely contributing to the prolonged effects of siRNA therapeutics [17]. In contrast, ASOs are single-stranded modified DNA molecules which complementarily bind to the target mRNA. The target mRNA function is then inhibited by different mechanisms, one of the most important ones being cleavage by RNase H1. Different from the mechanism of siRNA, mRNA cleavage occurs in a one-to-one stoichiometry [18].

The siRNA compound inclisiran inhibits PCSK9 function by preventing its production through intracellular binding and degradation of PCSK9 mRNA [19]. The siRNA is conjugated with the sugar *N*-acetylgalactosamine (GalNAc) that binds to the ASGPR receptor expressed on hepatocytes. This strategy mediates the cellular uptake and hepatic selectivity. The siRNA approach allows for twice-yearly dosing after the first two injections have been administered three months apart. The long-term lowering of LDL-C provides potential advantages with regard to medication adherence compared to the antibodies and daily oral lipid-lowering medications such as statins or ezetimibe. Inclisiran reduces LDL-C by about 50% [20,21]. The ongoing ORION-4 trial will provide definitive and quantitative information on the effects of intracellular inhibition of PCSK9 with regard to cardiovascular events, tolerability, and safety [22].

While targeting mRNA leads to temporary effects and does not introduce any changes in the genome, gene editing technologies such as the CRISPR/Cas system aim to introduce permanent genomic changes to alter gene function. An approach of editing the *PCSK9* gene appears as the logical next step in the development of PCSK9 inhibitors. Firstly, by targeting one step further upstream of the protein synthesis pathway (protein, mRNA, DNA) and secondly, by additionally reducing the frequency of administration to potentially once in a lifetime.

## 3. Genome Editing Using the CRISPR/Cas System

### 3.1. The CRISPR/Cas System as Part of Adaptive Bacterial Immunity

The CRISPR/Cas system is part of the adaptive immunity of bacteria and archaea [23]. The adaptivity to previously encountered aggressors such as viruses relies on specific nucleic acid sequences in the bacterial genome. The clustered regularly interspaced short palindromic repeats (“CRISPR”) designate repetitive elements within the bacterial genome, which are followed by short fragments of foreign nucleic acids of viral or plasmid origin, which become integrated after the organism was challenged by the foreign genetic material. The respective parts of bacterial DNA are transcribed to RNA which then contains complementary sequences to those of the previously invading viral or plasmid genetic material [24]. If previously encountered foreign DNA enters the bacterial cell and complementary binds to the CRISPR RNA, this complex is then guided to a nuclease (“Cas”, CRISPR-associated) which introduces a double strand break in the foreign DNA to silence it [25].

### 3.2. Application of the CRISPR/Cas System for Genome Editing

#### 3.2.1. Principle of Application

The potential to exploit the CRISPR/Cas system for genome editing by using a programmable RNA was discovered in 2012 by Jennifer A. Doudna and Emmanuelle Charpentier, who were awarded the Nobel Prize in Chemistry in 2020 for their work [26]. Since its discovery, use of the CRISPR/Cas system for genome editing has rapidly expanded, especially using the CRISPR/Cas9 system derived from *Streptococcus pyogenes* [23]. In CRISPR/Cas9 genome editing, only a Cas9 nuclease and a guide RNA (gRNA) are required. Both are discussed below. Current applications include, among others, the study of genetic diseases in cell and animal models to gain insights into pathophysiology, and analyses of the effects of genetic variants. Potential therapeutic opportunities include both the treatment of disease *caused* by specific mutations and the treatment of disease not primarily caused genetically, by *introducing* mutations [27].

**Figure 1:**
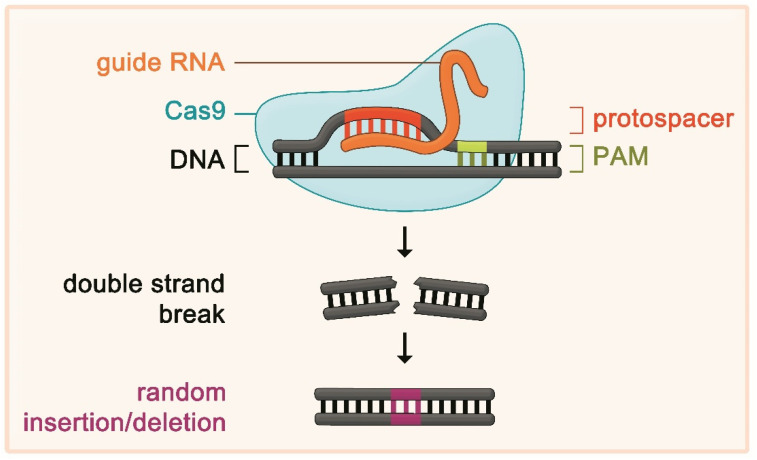
Shown is the principle of using the clustered regularly interspaced short palindromic repeat (CRISPR)/CRISPR-associated (Cas) system for genome editing. The programmable guide RNA (gRNA) complementary binds to a specific DNA sequence termed “protospacer”. The DNA–gRNA complex recruits a nuclease such as Cas9. The nuclease has to interact with a specific DNA sequence next to the protospacer termed “protospacer-adjacent motif” (PAM) in order to work. If these requirements are fulfilled, the nuclease introduces a double strand break in the DNA. Endogenous DNA repair mechanisms follow. These repair mechanisms are error-prone and lead to random insertions or deletions, which, in turn, render the gene and the encoded protein dysfunctional. In a further development, the catalytic domain of Cas9 has been deactivated and fused to a base editor, which does not introduce double strand breaks, but precise single-nucleotide changes.

In bacteria, two different kinds of RNA called “crRNA” and “tracrRNA” are necessary for the CRISPR/Cas system to facilitate cleavage of foreign DNA material. The crRNA carries the guide sequence directing the complex to a specific DNA site. To exploit the CRISPR/Cas system for intended genome editing, the prerequisite of the presence of two different RNAs could be circumvented by a major breakthrough which led to a considerable simplification of applicability: the development of a synthetic gRNA, which can take over the function of the two bacterial RNAs [26]. The gRNA directs the nuclease to a specific genomic site by Watson–Crick base pairing of the first 20 nucleotides of the gRNA with the target DNA sequence. The respective DNA sequence is called “protospacer” and needs to be situated next to a protospacer-adjacent motif (PAM), which interacts with the nuclease (e.g., the sequence of the PAM the *Streptococcus pyogenes* Cas9 interacts with is NGG, where “N” is any nucleotide, and “G” is guanine) [23]. By designing the 20-nucleotide sequence of the gRNA, the CRISPR/Cas9 system can be directed to virtually any 20 nucleotide-sequence which is located nearby a PAM. After the nuclease has introduced a double strand break in the target DNA, the endogenous DNA repair system aims to repair the break by different mechanisms. However, this process is error-prone and regularly leads to random insertions or deletions at the site of the break. This, in turn, can cause a shift of the reading frame and by this renders the gene dysfunctional [27] (Figure 1).

The Cas9 nuclease is the only protein required to induce the necessary breaks in the DNA. Many variations of the Cas9 nuclease have been discovered and many more Cas9 alternatives have been engineered. While the first discovered Cas9 proteins could only induce double strand breaks, various laboratories have employed base editors by using a catalytically inactive Cas9 protein coupled to a cytidine or adenosine deaminase [28]. Like Cas9, a base editor requires the presence of a gRNA that directs it to a specific DNA sequence—the protospacer—in order to work. Instead of introducing double strand breaks, the base editor leads to single-nucleotide changes within the protospacer, while at a specific position within the protospacer, peak base editing activity occurs [29]. For example, by using an adenine base editor, an adenine (A) (pairing to thymine (T) on the opposite DNA strand) at the peak editing position is deaminated by the base editor and thereby becomes inosine (I). The other strand is nicked. By nick repair, inosine pairs to cytosine (C) instead of T, and following this, inosine is replaced by guanin (G) to pair with C. As result, A–T has become G–C without the need for a double strand break [6].

#### 3.2.2. Advantages and Limitations of CRISPR/Cas Genome Editing

Use of the CRISPR/Cas system for genome editing has several advantages compared to other previously developed strategies, such as zinc-finger nucleases and transcription activator-like effector nucleases (TALEN). The aforementioned are based on protein-mediated DNA cleavage and require protein engineering to gain site specificity, while the programmable part of the CRISPR/Cas system (the gRNA) is relatively simple and inexpensive to design and produce. Furthermore, CRISPR/Cas allows the use of several different gRNAs in one vector at the same time to address different genomic sites [30,31].

There are, however, also restrictions to this approach that potentially limit its universal applicability. The most important concern are off-target effects, i.e., genome editing at sites other than the targeted one due to a sequence homology of the target and the off-target site. Off-target effects can lead to the unintended inhibition of other genes and potentially oncogenesis [32]. Although many approaches such as modification of the gRNA have been pursued to minimize the potential for off-target effects, off-target editing remains a major concern inherent to the methodologic principle and needs to be carefully addressed before any potential clinical application. This is of special importance, as the detection of off-target mutagenesis is challenging [33]. Other gene editing approaches which do not lead to double strand brakes and are considered less likely to cause off-target editing include the use of base editing (as discussed above) and prime editing; the latter principle is based on the use of Cas9 fused to a modified reverse transcriptase which is guided by an RNA template that serves as primer for reverse transcriptase and contains the edit sequence as extension of the gRNA [34]. Furthermore, although the gRNA can in principle be targeted at any genomic location, the activity of the associated nuclease depends on the presence of a PAM next to the targeted sequence. While this as a minor limitation for the three-nucleotide PAM of Cas9 of *Streptococcus pyogenes*, other bacteria’s PAMs are larger, more complex, and less frequently present in the genome. Strategies to overcome this limitation include the use of different Cas9 proteins and mutagenesis of the protein which influences its PAM specificity [35]. Moreover, to date, gene editing has to be considered an irrevocable treatment which, in case of adverse effects, cannot be discontinued as conventional drugs can [36]. Finally, current methods in genome editing are highly variable in their efficiency, depending on the genetic locus and the tissue. However, studies have shown that the efficiency of gene editing is relatively high in hepatocytes, which is important for the potential future treatment of dyslipidemias. In contrast, efficiency appears low, e.g., in cardiomyocytes [27].

## 4. Editing of *PCSK9* Using CRISPR/Cas

At the crossroads of a promising target gene such as *PCSK9* and the means of specific and potentially permanent gene editing, different research groups pursued editing *PCSK9*.

### 4.1. Initial In Vivo Studies in Mice

The first in vivo study in mice was published by Ding et al. [37]. The authors selected a gRNA targeting exon 1 of mouse *Pcsk9* and generated an adenovirus expressing this gRNA and Cas9. Three to four days after injection of the adenovirus, *Pcsk9* mutagenesis had occurred in >50%, resulting in substantially decreased PCSK9 levels and reductions in total cholesterol of 35–40%. No off-target effects were observed. This approach was, however, limited by the use of an adenovirus, which can evoke an immune response and does not appear optimal for application in humans [27]. In addition, the strategy leads to permanent expression of Cas9, which did not appear to be necessary since more than 50% of *Pcsk9* had been edited three days after administration. The use of an adeno-associated virus as preferable vector to treat humans was not possible as the *Streptococcus pyogenes* Cas9 mRNA together with the gRNA were too large to fit into this vector.

Wang et al. used a mouse model with humanized hepatocytes [38]. Again, an adenovirus was used as vector, carrying the genetic information of *Streptococcus pyogenes* Cas9 and a gRNA targeting exon 1 of *PCSK9*. Four days after injection of this vector, almost 50% mutagenesis of *PCSK9* and 52% reductions in human PCSK9 protein occurred. As the mice in this study were a chimeric liver-humanized model, mouse PCSK9 protein levels increased, probably as a compensatory mechanism, which the authors indicate as reason why no effects on total cholesterol levels were observed. Similar to the study by Ding et al., no relevant off-target mutagenesis was detected.

Using an adeno-associated virus instead of the adenovirus, Ran et al. employed a Cas9 orthologue from *Staphylococcus aureus* instead of the *Streptococcus pyogenes* Cas9 [39]. As the *Staphylococcus aureus* Cas9 is smaller, its genetic information fits in an adeno-associated virus vector together with a gRNA targeting mouse *Pcsk9*. One week after injection in mice, greater than 40% mutagenesis in *Pcsk9* was observed, and Pcsk9 protein and total cholesterol levels were significantly reduced by 95% and 40%, respectively.

### 4.2. In Vivo Studies in Non-Human Primates

To date, there are three reports on *PCSK9* gene editing in non-human primates.

#### 4.2.1. *PCSK9* Editing Using a Meganuclease

The first in vivo gene editing study in non-human primates was published by Wang et al., who reported the use of adeno-associated virus carrying the genetic information for a meganuclease [40]. The principle behind meganucleases has to be differentiated from the CRISPR/Cas system. Meganucleases are engineered from homing endonucleases (identified in yeasts) which introduce double strand breaks at specific sequences. Meganucleases are small and work with high specificity, but are considered more difficult to engineer compared to the CRISPR/Cas system. The DNA sequence specificity of the meganucleases is achieved by protein engineering, whereas the specificity of the CRISPR/Cas system relies on the sequence of the gRNA. The authors tested their approach in rhesus macaques and observed dose-dependent reductions in PCSK9 levels of up to 84% with LDL-C reductions of up to 60%. However, a relevant number of off-target sites were identified, where unintended editing occurred. Following detailed analyses of the off-target sites, the nuclease was re-engineered and was shown to cause less (but still detectable) off-target editing while maintaining LDL-lowering efficacy. The injections were accompanied by transient elevations of serum transaminases due to a T cell response. The treated non-human primates were followed for up to three years and showed stable reductions in serum PCSK9 and LDL-C [41]. The initially detected off-target editing remained stable over time.

#### 4.2.2. *PCSK9* Editing Using a Base Editor (I)

While the utilization of a meganuclease has the advantage of enabling the use of adeno-associated virus instead of adenovirus, the major downsides were the observed significant off-target editing and the immune response. In order to circumvent both of these problems, another option is the delivery of a CRISPR/Cas gene editing tool to its site of action by lipid nanoparticles. Conceptually, these nanoparticles contain the gRNA and, in addition, either the nuclease as a protein or the genetic material encoding the nuclease as plasmid or mRNA. In the latter case, the host ribosomal system transcribes the nuclease, and the injected mRNA as well as the nuclease are degraded over time. The transient expression of the nuclease appears sufficient to achieve sustainable genome editing. Severe immune responses are not expected because the nanoparticles do not contain any viral material [42].

Such nanoparticles were used in the second study on *PCSK9* editing in non-human primates. The study was conducted by Musunuru et al. and represents the most advanced published effort of in vivo editing of *PCSK9* to date [6]. The most important difference compared to previous studies is the use of a specific base editor that does not lead to double strand breaks of the DNA but to a highly specific exchange of one nucleotide. The study is the first report of successful in vivo use of a base editor in non-human primates. After an initial proof-of-principle study from the same group in which they tested the feasibility of a base editor in mice [43], the authors conducted a series of in vitro studies followed by studies in mice and cynomolgus monkeys (*Macaca fascicularis*) and employed an adenine base editor.

**Figure 2:**
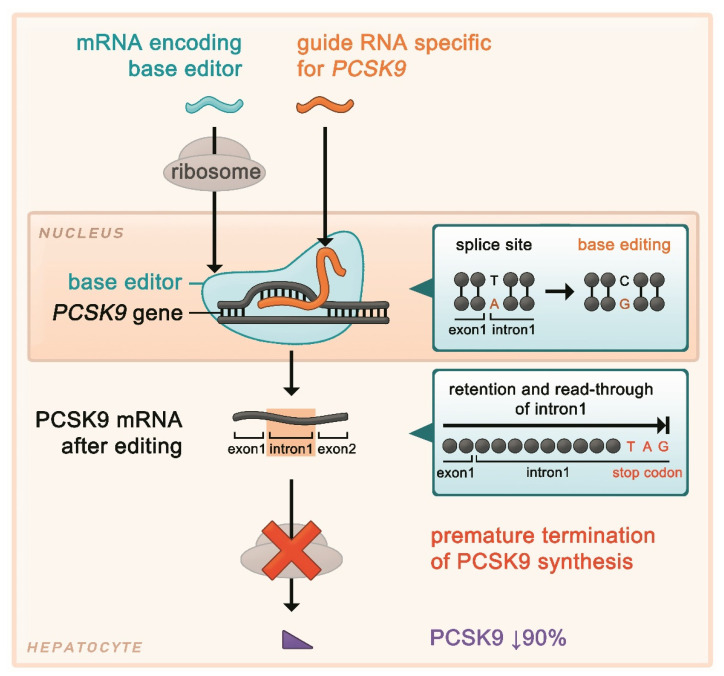
Genome editing of *PCSK9* using a base editor that introduces a single-nucleotide change at the splice site of exon 1 and intron 1. This results in retention of intron 1 in the final mRNA, which contains an in-frame stop codon, leading to premature termination of protein synthesis.

The authors selected and designed a gRNA that targets a *PCSK9* mRNA splice site. Splice sites are characterized by GT and AG sequences (donor and acceptor sites, respectively), and nucleotide exchanges at these sites may disrupt regular splicing of the precursor mRNA. After identifying several potential gRNAs which would theoretically direct the base editor to an A in a splice site of the precursor mRNA, in in vitro studies of human hepatocytes, a gRNA with a high level of base editing at the desired site, but no relevant editing activity at other genomic sites was selected. This gRNA directs the base editor to the splice site between exon 1 and intron 1 of the *PCSK9* gene. The transition of A to G at the splice site leads to its disruption, which has the effect of retention of intron 1 in the final mRNA. In intron 1, there is an in-frame stop codon with the sequence TAG, which leads to termination of PCSK9 protein synthesis after three amino acids (encoded by intron 1) have been added to those encoded by exon 1 (Figure 2).

Delivery of the gene editing tool was facilitated using lipid nanoparticles containing the chemically synthesized gRNA and in vitro transcribed mRNA encoding the base editor. In vitro studies of human hepatocytes revealed 60% base editing at the targeted splice site, and PCSK9 expression was reduced by 55%. Similar results were obtained in cynomolgus monkey hepatocytes, as the targeted sequence in the primate *PCSK9* orthologue is identical to that of the human one. Next, the authors went on to study their approach in mice, using a gRNA matching the *Pcsk9* mouse orthologue sequence. Base editing was observed in 70% of the liver (meaning that virtually all hepatocytes were affected, as around 70% of liver cells are hepatocytes).

Following this, cynomolgus monkeys were treated. The nanoparticles containing the two types of RNA were given intravenously. In two short-term studies with necropsy 24 h and two weeks after infusion, an average of 63% gene editing was observed. PCSK9 protein and LDL-C levels were reduced by 81% and 65%, respectively. Tissues other than liver were only minimally affected by base editing. Of note, the monkeys were pre-treated with dexamethasone, famotidine, and diphenhydramine. Still, a small, transient rise in liver transaminases was observed which normalized two weeks after infusion. In further investigations, this transient rise could be attributed to the lipid nanoparticles rather than to the RNAs they contained. All components of the infusion were cleared within two weeks. By comparing the selected gRNA with a human and cynomolgus monkey genomic library, the authors identified up to 67 relatively homologous genomic sites at risk for off-target editing The authors then investigated off-target editing at these sites using treated monkey hepatocytes and liver biopsies of treated monkeys, and found that off-target editing was very infrequent with the most predominant off-target editing site showing less than 1% editing. This most predominant off-target editing site was in the monkey *PCSK9* gene, which differs from the human orthologue. Human hepatocytes were also tested, and no off-target editing was observed.

The final and probably most important part of the study is a still ongoing long-term study with treated monkeys with a follow-up of up to eight months. Here, sustained effects of *PCSK9* editing with reductions in PCSK9 and LDL-C levels by a mean of 90% and 60%, respectively, were observed. Comparably to pharmacologic PCSK9 inhibition, a 35% reduction in lipoprotein(a) concentration was observed. After initial and resolved liver enzyme elevations similar to the short-term study, no late increases in liver function tests occurred.

As the authors discuss in their study [6], their approach may have several advantages compared to using a meganuclease. Regarding efficacy, the frequency of achieved *PCSK9* editing was higher. Furthermore, a single nucleotide exchange represents a small intervention compared to inducing a DNA double strand break and integrating viral genome in the host genome. Additionally, the rise in aminotransferases was more pronounced and sustained (up to months) in the study of Wang et al., while after the lipid nanoparticle infusion, only a short-term response was observed which was due to the lipid nanoparticle itself rather than its content. Lastly, Musunuru et al. did not observe any off-target editing, while Wang et al. observed significant off-target activity.

#### 4.2.3. *PCSK9* Editing Using a Base Editor (II)

The third study on *PCSK9* editing in non-human primates was conducted by Rothgangl et al. [44]. Similarly to the study of Musunuru et al. [6], the authors used an adenine base editor which was delivered by a lipid nanoparticle, containing the base editor mRNA and a gRNA. The gRNA targets the splice donor site of intron 1, and was selected in in vitro studies among several gRNA candidates that target different *PCSK9* splice sites. The final selected gRNA led to the highest rate of base editing and substantial reductions in PCSK9 mRNA and protein levels. After successful studies in mice, the authors treated cynomolgus monkeys with different doses (in part with repeated administration) of the mRNA and gRNA-containing lipid nanoparticles. The monkeys were euthanized at day 29. Treatment was accompanied by transient rises in aminotransferases, which were resolved at day 7 after infusion. Base editing occurred in a mean of 26%, with reductions in PCSK9 protein by 32% and LDL-C by 14%. Because repeated dosing did not increase the rate of base editing, the authors investigated for a potential immune response and found that the re-treated animals had formed IgG against the base editor. No off-target editing was observed.

Compared to the study of Musunuru et al. [6], in the study of Rothgangl et al. [44], a similar approach was used; however, the follow-up was shorter and the efficacy of base editing and effects on PCSK9 and LDL-C were less pronounced. Repeated dosing was unfortunately not evaluated by the study of Musunuru et al., and the identification of the IgG-mediated response by Rothgangl et al. warrants further investigation into the immune response to base editors.

The key data of the three studies described above are compared in Table 1.

## 5. Discussion

Only 15 years have passed between the identification of PCSK9 as regulator in lipid metabolism to the proof of beneficial effects of therapeutic PCSK9 inhibition in humans. The discovery that the CRISPR/Cas system can be used as a tool to edit eukaryotic genomes is even more recent. The combination of a promising treatment target and a highly specific, potentially one-time treatment modality provides important insights and great potential. Several aspects should be noted:

Firstly, established lipid-lowering medications such as statins or ezetimibe have to be taken daily. The PCSK9 inhibitors, or more recently, the siRNA inclisiran are administered every two to four weeks or biannually, respectively. While there has been remarkable progress in the treatment of dyslipidemias in the last decades, medication adherence remains relevant to mortality [45]. Hypothesizing that the “once-and-done approach” [6] of Musunuru et al. will be effective and safe in humans, this would resolve the issue of medication adherence. The observed reduction in LDL-C levels of about 60% is comparable to what can be achieved by statins or PCSK9 antibodies [46], and the notion of sustained reductions in LDL-C might offer additional benefits as to the cumulative effects of LDL-C on atherosclerosis [47]. Another potential advantage is that like PCSK9 antibodies and the siRNA inclisiran, for CRISPR/Cas gene editing, no interactions with other drugs or restrictions such as reduced kidney function are expected.

Secondly, the data of Musunuru et al. appear promising in different ways. Genome editing occurred 1. virtually organ-specific, 2. specific for the genomic site, 3. with no relevant off-target mutagenesis, 4. with only a minor modification of the host genome without relying on random insertions or deletions after a double strand DNA break, and 5. without integration of viral genome. All of the injected components were shown to be rapidly degraded. However, data on long-term safety in non-human primates are needed before potential application in humans.

Thirdly, the current evidence on *PCSK9* editing opens the stage for other genetically validated treatment targets in dyslipidemia such as lipoprotein(a), angiopoietin-like 3, and apolipoprotein CIII which can already be silenced with antisense-oligonucleotides and siRNA [18]. Early attempts to addressing these targets appear promising [36,48].

Fourthly, one should keep in mind that despite the conceptual elegance and apparent safety, long-term efficacy and safety need to be demonstrated before any clinical application. Since the current methods lead to (presumably) irreversible alterations in the genome, this implicates that the efficacy will last for a lifetime, without a simple option to discontinue treatment. This is in sharp contrast to the more flexible administration of PCSK9 monoclonal antibodies or siRNA, with the option to discontinue treatment. Moreover, a potential re-edit back to the original genomic code introduces additional risk of off-target editing. Furthermore, although *PCSK9* loss-of-function carriers do not appear to experience adverse health issues, the possibility exists that therapeutically induced *PCSK9* knockout may cause adverse effects. For example, it was shown that *PCSK9* knockout mice develop hepatic steatosis after high-fat diet related to increased expression of CD36 on hepatocytes [49]. To fully estimate the clinical importance of this notion, the effects of different diets need to be investigated while taking in mind that *Macaca fascicularis*, investigated in two of the mentioned studies [6,44], are vegetarians. The irreversible alterations in the genome thus also implicate that phase 1 clinical trials will have to commence in patients rather than in healthy volunteers. Moreover, the lipid nanoparticles currently seem to elicit a significant immune response. An option to increase specificity to the liver and possibly reduce the immune response may be the incorporation of GalNAc molecules into the lipid nanoparticle surface [50]. Another concern is the identification of target patients. The efficacy of *PCSK9* gene therapy might be limited in FH patients that exhibit an *LDLR* mutation, since these patients already have affected LDL-C uptake by expressing less LDL receptors; however, clinical trials of PCSK9 antibodies in patients with heterozygous FH showed comparable LDL-lowering efficacy as in patients without FH [14,51]. Gene editing of *LDLR* itself in these patients might be another possible step. These concerns will have to be addressed in future studies.

Finally, genome editing is fundamentally different from other established pharmacologic treatment options in that it aims to induce irreversible genomic changes. While edits in germline cells could be passed to offspring, the case is different for the approaches discussed here, in which only somatic cells were targeted. While changing only one single person’s genome is generally considered more tolerable, before any routine clinical application, thoughtful and critical ethical debate should precede [52].

## 6. Conclusions

The use of the CRISPR/Cas system has expanded to all disciplines of medicine. While monogenetic disorders appear as an obvious primary goal of the gene editing approach [53], gene and base editing technologies may eventually become part of the therapeutic armamentarium for more common conditions such as dyslipidemia. In theory, a one-time application at young age could lead to a sustained reduction in LDL-C and dramatically reduced cardiovascular risk [54], which opens the stage for true primary prevention of atherosclerosis.

## Figures and Tables

**Table 1:** Comparison of studies on *PCSK9* editing in non-human primates.

First Author, *Journal* (Year of Publication) [Reference]	Wang, *Nature Biotechnology* (2018/2021) [40,41]	Musunuru, *Nature* (2021) [6]	Rothgangl, *Nature Biotechnology* (2021) [44]
Non-human primate	Rhesus macaques (*Macaca mulatta*)	Cynomolgus macaques (*Macaca fascicularis)*	Cynomolgus macaques (*Macaca fascicularis*)
Delivery method	Adeno-associated virus transducing genetic information for the meganuclease	Lipid nanoparticle containing gRNA and base editor mRNA	Lipid nanoparticle containing gRNA and base editor mRNA
Genome editing technology	Meganuclease, induction of double strand breaks at specific site, random deletions/insertions after repair	Adenine base editor, specific single-nucleotide exchange	Adenine base editor, specific single-nucleotide exchange
Length of follow-up	Up to 3 years	8 months	29 days
*PCSK9* editing *	Up to 46%	66%	Max. 34%, mean 26%
Reduction PCSK9	Up to 84%	90%	32%
Reduction LDL-C	Up to 60%	60%	14%
Off-target effects	Up to 629 detected off-target DNA cleavages	None detected	None detected
Immune response	T cell response	None detected	Humoral immune response against base editor

* The liver contains about 70% hepatocytes, which is why 70% editing means DNA editing in virtually all hepatocytes [6,40]. PCSK9: proprotein convertase subtilisin/kexin type 9; LDL-C: low-density lipoprotein cholesterol.
